# Multimodal temporal dissociation in anti-GABA_A_ receptor encephalitis: functional recovery precedes structural resolution-a case report

**DOI:** 10.3389/fimmu.2026.1857947

**Published:** 2026-07-27

**Authors:** Yi Huang, Jiayue Li, Qi Gao, Minjue Gu, Qipeng Jin, Qingyin Kong, Lingpeng Meng, Jianan Qian, Ran Chen, Yiru Wang, Yiyi Zhang

**Affiliations:** Department of Critical Care Medicine, Longhua Hospital, Shanghai University of Traditional Chinese Medicine, Shanghai, China

**Keywords:** autoimmune encephalitis, electroencephalography, immunomodulation, magnetic resonance imaging, receptors, GABA-A

## Abstract

**Background:**

Anti-γ-aminobutyric acid A receptor (GABA_A_ receptor) encephalitis is a rare but severe subtype of autoimmune encephalitis (AE), typically characterized by refractory seizures and rapidly progressive neurological dysfunction. Although structural abnormalities on magnetic resonance imaging (MRI) are frequently observed, the temporal relationships among clinical manifestations, electrophysiological changes, immune activity, and neuroimaging findings remain insufficiently characterized.

**Case presentation:**

We report a 61-year-old man with anti-GABA_A_ receptor encephalitis who underwent longitudinal multimodal evaluation. In the early stage, the patient presented with nonspecific vestibular symptoms accompanied by subtle cortical abnormalities on MRI. The disease rapidly progressed to status epilepticus and cognitive impairment. Electroencephalography (EEG) revealed widespread epileptiform discharges, while cerebrospinal fluid (CSF) analysis demonstrated inflammatory changes, and both serum and CSF were positive for anti-GABA_A_ receptor antibodies. Following timely immunotherapy, the patient exhibited rapid clinical improvement, marked attenuation of epileptiform activity, and subsequent antibody negativization. Notably, structural MRI abnormalities persisted and resolved more slowly than clinical and electrophysiological recovery. Translocator protein positron emission tomography (TSPO-PET) using the 18F-DPA714 demonstrated increased tracer uptake in the bilateral temporo-parieto-occipital cortices, indicating persistent neuroinflammation despite clinical improvement.

**Conclusions:**

This case provides longitudinal multimodal observations supporting the concept that anti-GABA_A_ receptor encephalitis may represent a dynamic and potentially reversible disorder of network hyperexcitability rather than a purely structural inflammatory injury. The temporal dissociation between functional recovery and delayed structural resolution highlights the critical value of multimodal monitoring, particularly EEG and immunological biomarkers, in assessing disease activity and guiding immunotherapy.

## 1 Introduction

In recent years, advances in neuroimmunology and diagnostic technologies have led to the recognition of an increasing number of autoimmune encephalitis (AE) cases, most of which are associated with antibodies targeting neuronal surface antigens or synaptic proteins (1–3). AE is now regarded as a potentially treatable and reversible neurological disorder. Prompt recognition and early immunotherapy are critical because timely treatment is associated with substantial neurological recovery, whereas delayed diagnosis may result in irreversible neurological deficits (1, 4).

Among the various subtypes of AE, anti–γ-aminobutyric acid A receptor (GABA_A_ receptor) encephalitis represents a particularly rare form. GABA_A_ receptor is a key inhibitory neurotransmitter receptor in the central nervous system, functioning as a ligand-gated chloride channel that maintains inhibitory synaptic transmission and neural network stability. Autoantibodies targeting GABA_A_ receptor disrupt receptor function, leading to neuronal disinhibition and network hyperexcitability, which manifest clinically as seizures, cognitive impairment, and behavioral disturbances (5). Neuroimaging studies indicate that approximately 80% of patients exhibit abnormal magnetic resonance imaging(MRI) findings, typically presenting as multifocal cortical and subcortical T2/FLAIR hyperintensities, most commonly involving the temporal and frontal lobes (6). However, existing literature has largely focused on cross-sectional imaging features, while the temporal relationships between MRI abnormalities and clinical, electrophysiological, and immunological changes remain poorly defined.

Despite growing recognition of this disorder, fewer than one hundred cases have been reported worldwide since its first description in 2014, and its clinical and radiological spectrum remains incompletely characterized (7–9). Recent longitudinal cohort studies have highlighted the dynamic evolution of MRI abnormalities in anti-GABA_A_ receptor encephalitis (10), whereas the temporal interplay among clinical symptoms, electrophysiological activity, immune responses, and neuroimaging abnormalities has rarely been systematically investigated. Increasing evidence suggests that AE may fundamentally represent a disorder of neural network dysfunction rather than purely structural inflammatory injury (11), and that discrepancies between multimodal assessments may occur at different stages of the disease.

Of particular importance, structural MRI changes may lag behind functional and immunological abnormalities, potentially leading to diagnostic uncertainty in the early stages and delaying timely initiation of immunotherapy. Moreover, the temporal sequence of positivity across different diagnostic modalities has not been systematically characterized, limiting the identification of optimal diagnostic windows.

Here, we report a patient with anti-GABAA receptor encephalitis who underwent longitudinal multimodal evaluation to characterize the temporal evolution of clinical, electrophysiological, immunological, and structural imaging findings. This case not only provides insight into the dynamic evolution of this rare disease but also highlights the value of multimodal evaluation in improving early recognition and optimizing the timing of immunotherapy.

## 2 Case presentation

### 2.1 Patient information

A 61-year-old man was admitted to our hospital with a history of persistent dizziness for more than two months, accompanied by headache and progressive cognitive slowing for three days prior to admission.

His past medical history included thyroid nodules, grade-1 hypertension with very high cardiovascular risk, and left vertebral artery stenosis. He denied any prior neurological disorders, autoimmune diseases, malignancy, drug allergies, or relevant family history.

### 2.2 Initial presentation

**Figure 1:**
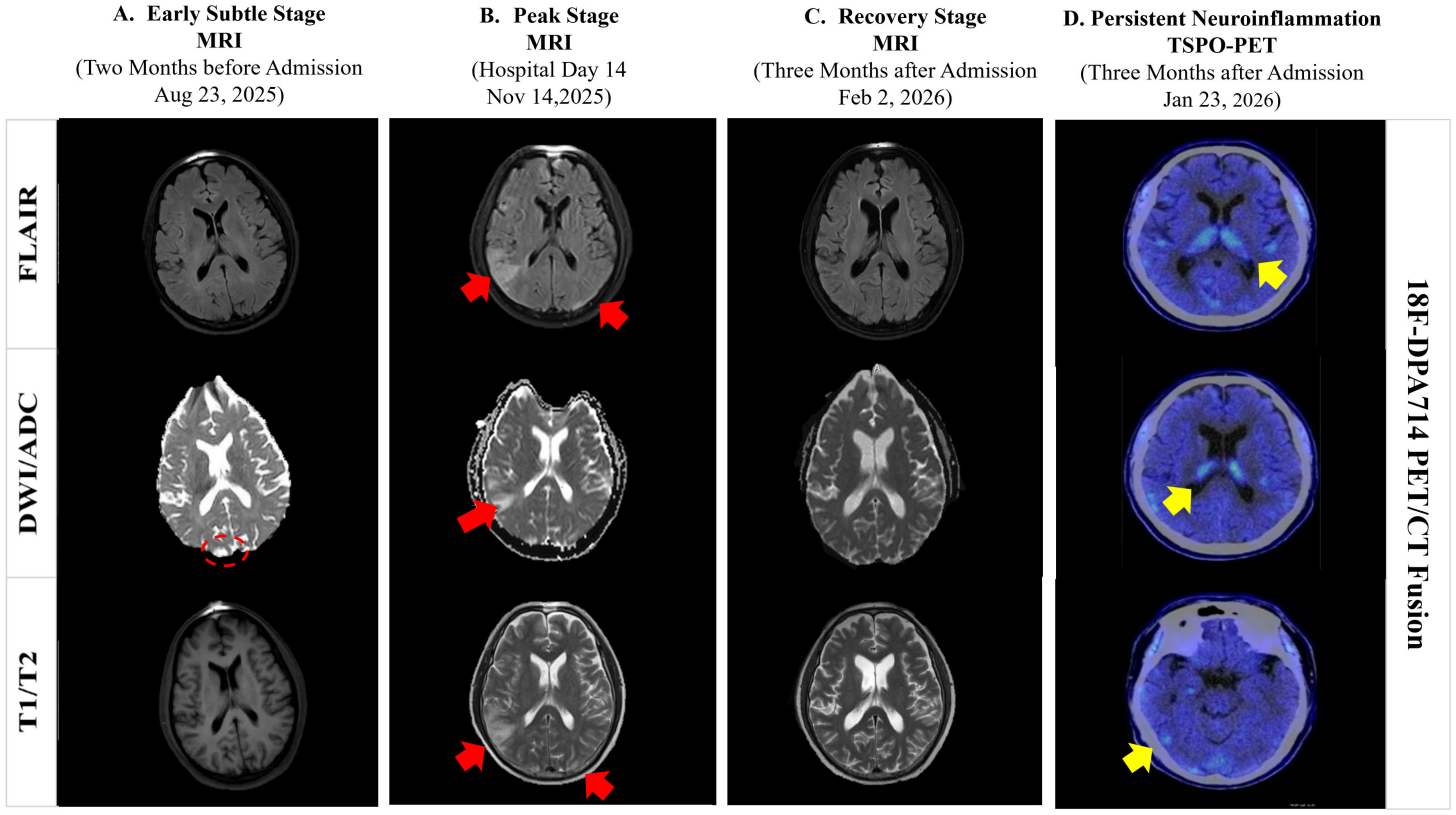
Multistage neuroimaging evolution in anti-GABA_A_ receptor encephalitis. Representative structural and molecular neuroimaging findings across different stages of the disease course. **(A)** Early stage (Two Months before Admission, Aug 23, 2025). Brain MRI shows a subtle focal cortical-subcortical signal abnormality in the right parieto-occipital region with mild hyperintensity on T2-FLAIR without diffusion restriction or contrast enhancement. The dashed circle highlights this small and nonspecific lesion. **(B)** Peak disease stage (Hospital Day 14, Nov 14, 2025). Brain MRI demonstrates extensive bilateral cortical-subcortical hyperintensities on T2-FLAIR and T2-weighted sequences, consistent with diffuse inflammatory involvement in AE. Red arrows indicate representative areas of cortical-subcortical signal abnormality. **(C)** Recovery stage (Three Months after Admission, Feb 2, 2026). Follow-up MRI shows near-complete resolution of the previously observed abnormalities. **(D)** Persistent neuroinflammation on TSPO-PET (Three months after admission). 18F-DPA714 PET/CT fusion images demonstrate persistent increased tracer uptake in the bilateral temporo-parieto-occipital cortices despite substantial structural recovery on MRI. Yellow arrows indicate representative regions of increased TSPO tracer uptake, consistent with persistent neuroinflammatory activity.

Approximately two months before admission, the patient developed recurrent dizziness without an obvious precipitating factor, accompanied by nausea and vomiting, but without focal neurological deficits. Brain contrast-enhanced MRI suggested a possible small ischemic lesion in the right parietal lobe (**Figure 1**). Computed tomography angiography (CTA) revealed vertebral artery atherosclerotic plaque formation with stenosis.

Based on these findings, the initial clinical diagnoses included vestibular neuritis or vertebrobasilar insufficiency. The patient received symptomatic treatment with dexamethasone, antiplatelet therapy, and lipid-lowering agents. Although symptoms partially improved, intermittent dizziness persisted.

Several days before admission, family members noted progressive cognitive slowing accompanied by persistent headache and nausea, prompting hospital admission for further evaluation. These initial diagnoses may have masked the underlying autoimmune process and contributed to a delay in definitive diagnosis.

### 2.3 Neurological deterioration

**Figure 2:**
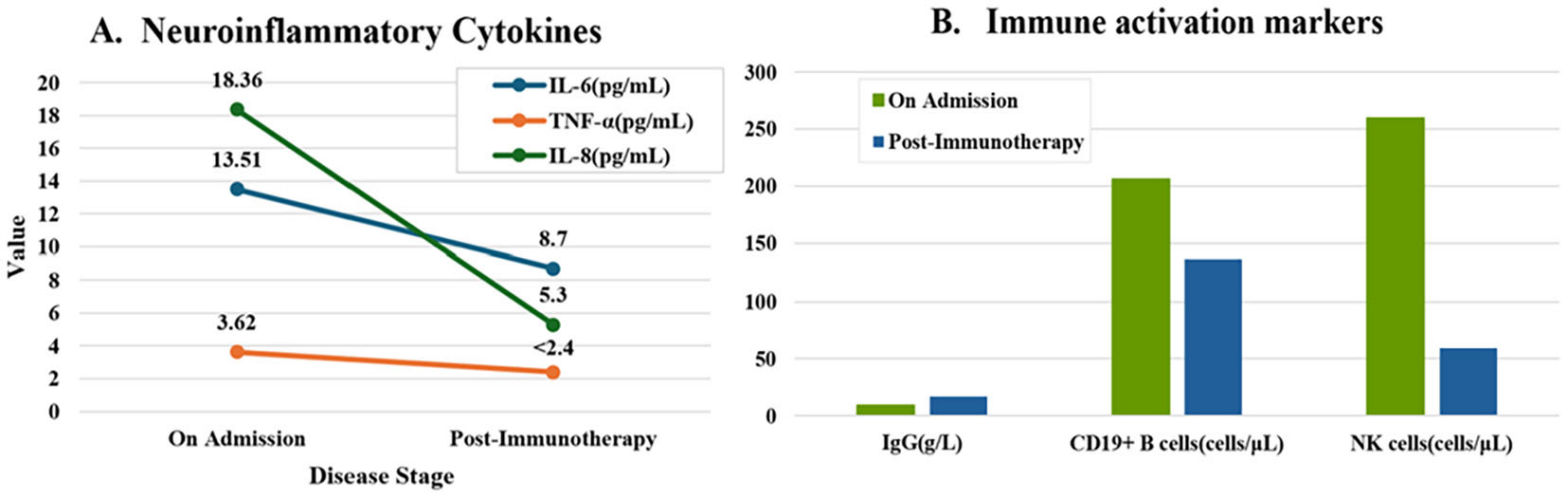
Longitudinal changes in peripheral immunological biomarkers during disease progression and treatment. **(A)** Neuroinflammatory cytokines, particularly IL-6 and IL-8, are markedly elevated during the acute phase and decline following immunotherapy, paralleling clinical recovery. **(B)** Immune profile analysis demonstrates increased IgG levels after treatment, accompanied by reduced proportions of NK cells and B cells, suggesting dynamic immune remodeling during disease recovery.

After admission, a comprehensive diagnostic workup was performed to exclude infectious encephalitis (including herpes simplex virus and other common viral encephalitides), bacterial central nervous system infection, thyroid dysfunction, systemic autoimmune diseases, malignancy, and other potential causes of encephalopathy. Relevant laboratory investigations are summarized in **Supplementary Table 1**, whereas the dynamic changes in peripheral immunological biomarkers are presented in **Figure 2**.

On Hospital Day 2, the patient experienced abrupt neurological deterioration, characterized by impaired consciousness and generalized convulsions. Despite treatment with antiepileptic medications, seizures remained refractory. He was therefore transferred to the intensive care unit (ICU), where endotracheal intubation and mechanical ventilation were initiated.

**Figure 3:**
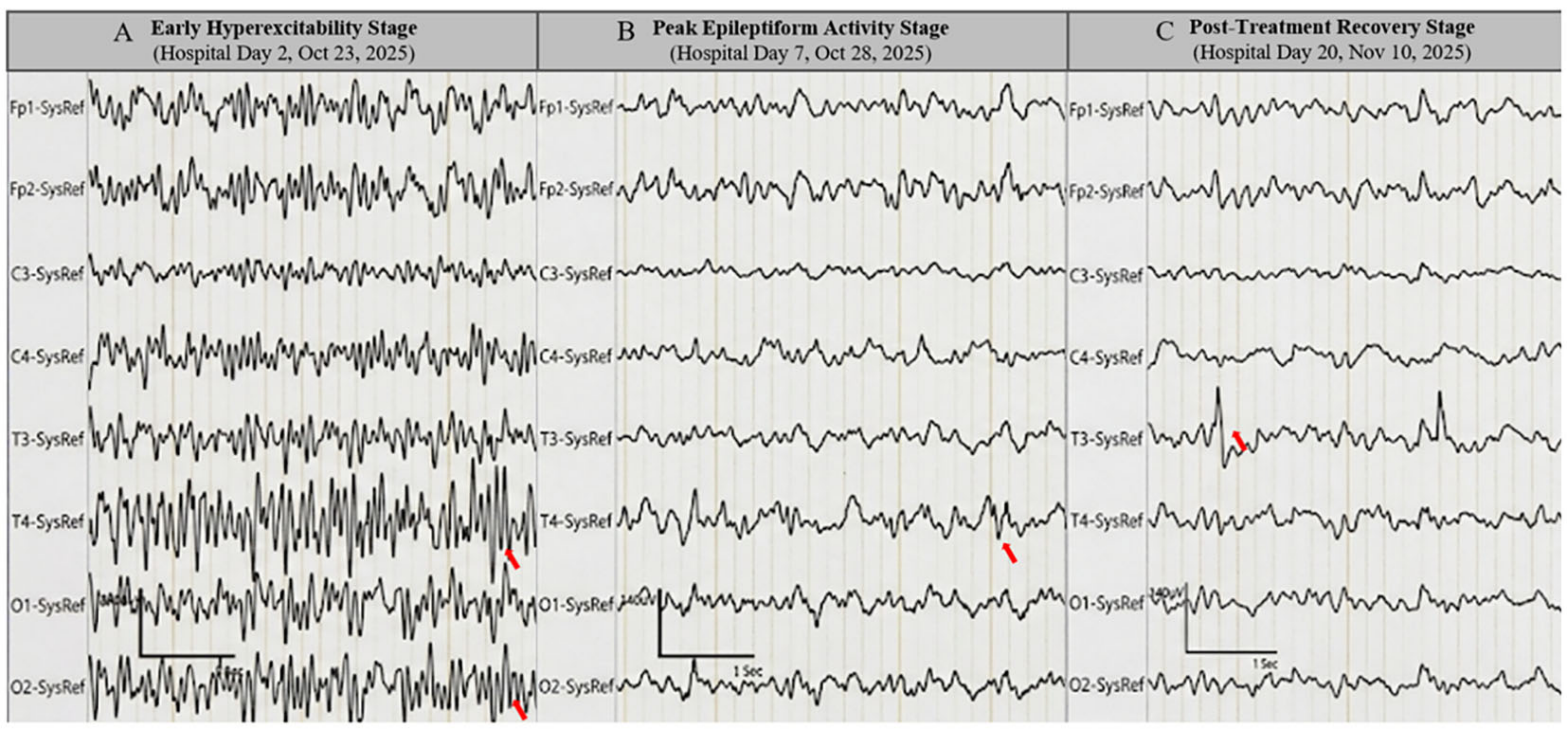
Dynamic electroencephalographic evolution of cortical hyperexcitability in anti-GABA_A_ receptor encephalitis. Representative EEG recordings obtained at different stages of the disease course. **(A)** Early hyperexcitability stage (Hospital Day 2, Oct 23, 2025). EEG demonstrates medium-to-high amplitude theta activity with frequent focal epileptiform discharges, predominantly in the right temporo-occipital region. The arrow indicates a high-amplitude spike-and-slow-wave complex, suggesting early cortical disinhibition. **(B)** Peak disease stage (Hospital Day 7, Oct 28, 2025). Persistent epileptiform activity is present, characterized by medium-to-high amplitude theta activity with recurrent spike-and-slow-wave discharges. The arrow indicates clusters of synchronized epileptiform discharges reflecting widespread cortical hyperexcitability. **(C)** Recovery stage following immunotherapy (Hospital Day 20, Nov 10, 2025). Background rhythms gradually normalize and epileptiform discharges markedly decrease. The arrow indicates residual focal sharp waves, suggesting partial electrophysiological recovery with persistent localized cortical irritability. EEG acquisition parameters are described in the Technical Details section.

Repeat head Computed Tomography(CT) on the same day revealed suspected new ischemic-like lesions in the right temporal, parietal, and occipital lobes. EEG demonstrated diffuse theta activity in both hemispheres with abundant epileptiform discharges, predominantly in the right temporo-occipital region (**Figure 3**).

### 2.4 Diagnostic workup

On Hospital Day 3, a lumbar puncture demonstrated inflammatory cerebrospinal fluid (CSF) findings: cell count: 13 × 10^6^/L; white blood cell count: 0; protein: 514 mg/L; oligoclonal bands: type IV pattern. AE antibody testing showed positive anti-GABA_A_ receptor antibodies in both serum (endpoint titer 1:10) and CSF (endpoint titer 1:1) (**Table 1**; **Supplementary Figure 1**). The CSF tissue-based assay (TBA) was positive (**Table 2**; **Supplementary Figure 1**), while demyelinating antibodies and paraneoplastic antibodies were negative. Antibody panel results are summarized in **Supplementary Table 2**.

**Table 1:** Dynamic cerebrospinal fluid and serum findings.

Date	Specimen	CSF (cells/μL)	WBC (cells/μL)	Protein (mg/L)	OCB pattern	Anti-GABAA receptor antibody	Tissue-based assay
Hospital Day 3	CSF	13	0	514	Type IV	Positive (1:1)	Positive
	Serum	–	–	–	–	Positive (1:10)	–
Hospital Day 17	CSF	–	–	–	–	Negative	–
	Serum	–	–	–	–	Negative	–
Hospital Day 36	CSF	–	6	697	Type IV	Negative	–
	Serum	–	–	–	–	Negative	–

**Table 2:** Cerebrospinal fluid tissue-based assay (TBA).

Date	Specimen	Region	Result	Titer	Interpretation
Hospital Day 3	CSF	Cerebral cortex	Negative	–	No specific fluorescence
	CSF	Hippocampus	Negative	–	No specific fluorescence
	CSF	Cerebellum	Positive	1:1	Dot-like fluorescence within the cerebellar granular layer, consistent with synaptic staining.

According to the 2016 Graus diagnostic criteria (3), the patient fulfilled the criteria for possible AE based on the subacute onset of cognitive impairment, new-onset refractory seizures, inflammatory CSF findings, supportive EEG abnormalities, and exclusion of alternative diagnoses. Autoimmune antibody testing further demonstrated anti-GABA_A_ receptor antibodies in both serum (endpoint titer 1:10) and CSF (endpoint titer 1:1). In addition, the CSF TBA demonstrated characteristic cerebellar granular layer (synaptic) staining, providing supportive evidence for neuronal surface autoimmunity. These findings established the diagnosis of anti-GABA_A_ receptor encephalitis.

### 2.5 Treatment and clinical course

Following multidisciplinary consultation, intensive immunotherapy was initiated, including high-dose intravenous methylprednisolone(1000mg/day for 3days) followed by gradual tapering, plasma exchange, and intravenous immunoglobulin (IVIG, 32.5g/day from Hospital Day 6 to 10 for 5 days followed by gradual dose reduction), together with antiepileptic and supportive treatments.

During the acute phase, a total of four plasma exchange procedures were performed: therapeutic plasma exchange (TPE) on Hospital Day 6, double filtration plasmapheresis (DFPP) on Hospital Day 8 and 13, and repeat TPE on Hospital Day 25.

After the initial TPE, the patient exhibited transient improvement in consciousness, with brief eye opening. Considering plasma resource utilization and the desire to minimize the loss of small molecular components, subsequent plasma exchange sessions were performed using DFPP.

However, during DFPP treatment, the patient’s neurological condition progressively deteriorated. EEG on Hospital Day 13 showed a marked expansion of epileptiform discharges compared with Hospital Day 7, with widespread rhythmic high-amplitude spike-and-wave activity. On Hospital Day 14, the patient developed another generalized tonic-clonic seizure.

Clinical improvement was not observed during DFPP treatment. Given the unsatisfactory clinical response, DFPP was discontinued, and immunotherapy was continued primarily with corticosteroids and IVIG.

Subsequently, the patient’s neurological function gradually improved. CSF re-examination on Hospital Day 17 showed negativization of anti-GABA_A_ receptor antibodies (**Table 1**). On Hospital Day 20, EEG demonstrated a marked reduction in epileptiform discharges with restoration of a symmetric background rhythm.

However, brain MRI on Hospital Day 24 still revealed multifocal cortical and subcortical abnormalities, consistent with typical imaging features of anti-GABA_A_ receptor encephalitis (**Figure 1**).

Following repeat TPE on Hospital Day 25, the patient showed marked improvement in consciousness, a reduction in seizure frequency, and gradual recovery of responsiveness to simple commands, further raising the possibility of different therapeutic responses between plasma exchange modalities.

### 2.6 Further management and follow-up

After invasive mechanical ventilation for 25 days and a total ICU stay of 28 days, the patient was transferred to the Department of Neurology for further treatment. Around Hospital Days 29-30, follow-up MRI continued to indicate ongoing inflammatory activity, and additional plasma exchange was performed along with continued immunotherapy.

On Hospital Day 36, repeat CSF analysis confirmed persistent negativity of anti-GABA_A_ receptor antibodies (**Table 1**). Rituximab therapy was subsequently initiated, combined with oral prednisone and antiepileptic medications. Whole-body Positron Emission Tomography/Computed Tomography (PET/CT) screening revealed no evidence of malignancy.

With continued treatment, the patient showed significant clinical improvement: no further seizures occurred, consciousness was fully restored, speech was fluent, and muscle strength improved to Medical Research Council grade 4 in all limbs, with only mild tremor in both hands. Corticosteroids were gradually tapered, and the tracheostomy tube was successfully removed.

Notably, approximately three months after admission, a Translocator Protein Positron Emission Tomography(TSPO-PET) scan demonstrated persistent tracer uptake, which may reflect ongoing neuroinflammatory processes in the bilateral temporo-parieto-occipital cortices. Approximately three and a half months after admission, in contrast, follow-up MRI showed near-complete resolution of the inflammatory lesions, with only residual small ischemic changes.

This dissociation between persistent neuroinflammation on molecular imaging and structural recovery on MRI further highlights the temporal mismatch between functional and structural recovery in anti-GABA_A_ receptor encephalitis.

**Figure 4:**
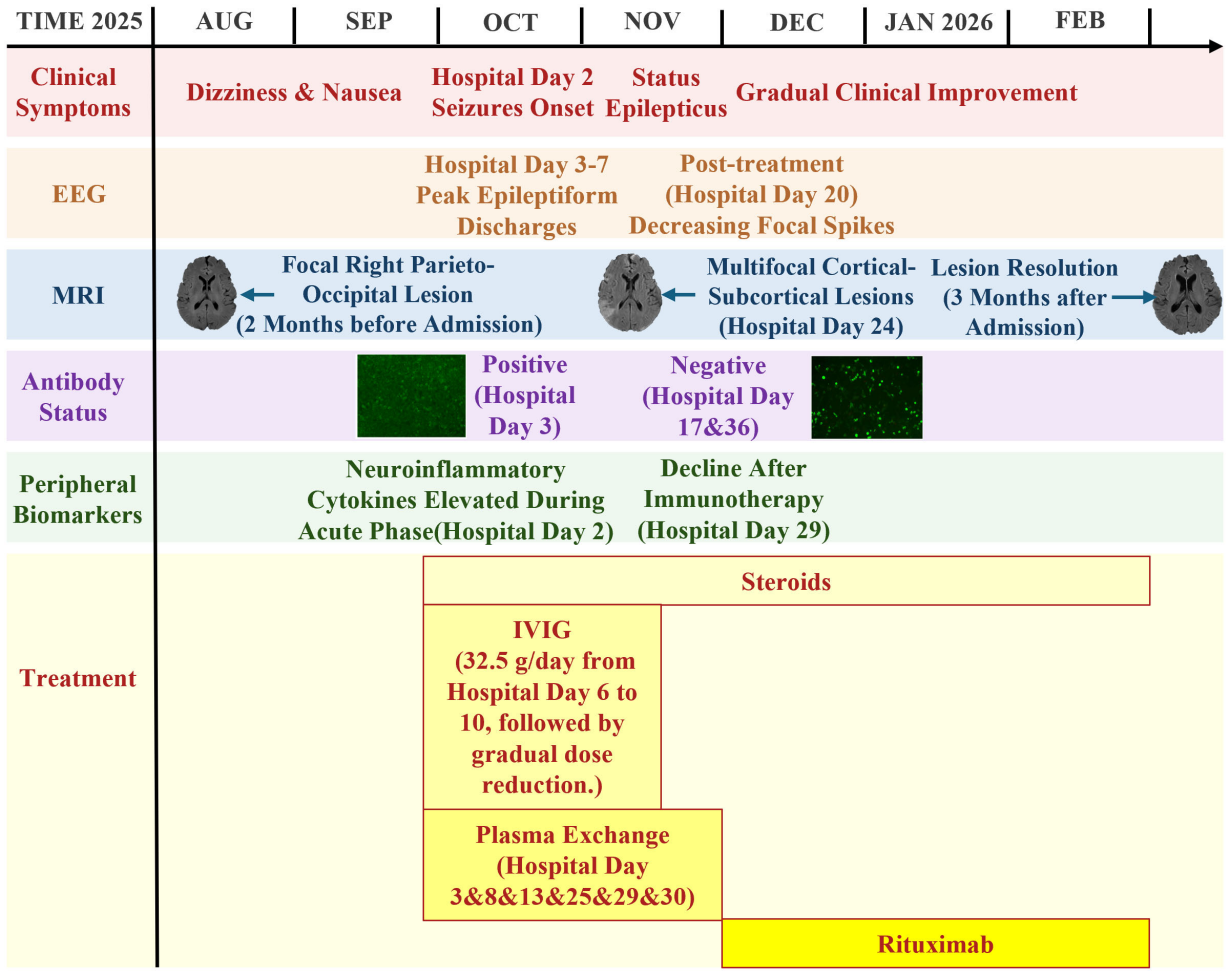
Integrated timeline of multimodal disease evolution. Schematic overview of the patient’s disease course from the prodromal phase to recovery, integrating clinical manifestations, laboratory biomarkers, neuroimaging findings, electroencephalographic changes, antibody status, and immunotherapeutic interventions. The image illustrates the temporal relationships among multimodal biomarkers, highlighting that electrophysiological and immunological improvements preceded structural MRI recovery.

The patient continued maintenance therapy with rituximab during follow-up (**Figure 4**).

## 3 Discussion

### 3.1 Clinical and radiological characteristics of anti-GABA_A_ receptor encephalitis

Anti-GABA_A_ receptor encephalitis is a rare but potentially rapidly progressive subtype of AE in which early diagnosis remains challenging despite its well-established immunological basis (3, 12). Neuroimaging studies have identified relatively characteristic MRI features in anti-GABA_A_ receptor encephalitis, typically presenting as multifocal, often confluent T2/FLAIR hyperintensities involving cortical and/or subcortical regions. These lesions frequently extend across cortical gray matter and adjacent subcortical white matter, forming a characteristic cortical-subcortical pattern (7, 13–15). Previous studies have suggested that these imaging abnormalities may primarily reflect cytotoxic edema secondary to cortical neuronal disinhibition (16–18).

Recently, Papi et al. reported the longitudinal MRI evolution of 33 patients with anti-GABA_A_ receptor encephalitis, demonstrating that imaging abnormalities evolve dynamically during disease progression and recovery (10). However, the dynamic evolution of these imaging findings and their relationship with clinical manifestations and immunological activity remain insufficiently characterized.

In the present study, longitudinal multimodal follow-up of a patient with anti-GABA_A_ receptor encephalitis enabled systematic characterization of the temporal relationships among clinical symptoms, neuroimaging changes, electrophysiological abnormalities, and immunological markers throughout disease onset, progression, and treatment. These observations provide longitudinal multimodal evidence that complements previous imaging-based studies and further illustrate the dynamic evolution of anti-GABA_A_ receptor encephalitis.

### 3.2 Temporal evolution of neuroimaging and its diagnostic implications

In the present case, the temporal evolution of neuroimaging findings provided important insights into the diagnostic process of anti-GABA_A_ receptor encephalitis.

At the early stage of the disease, the patient presented only with nonspecific vestibular symptoms, such as dizziness and nausea, without overt seizures or cognitive impairment. The initial brain MRI (August 23) demonstrated only subtle focal cortical-subcortical signal abnormalities, without diffusion restriction or contrast enhancement, and lacked the typical multifocal or limbic involvement commonly associated with AE. As a result, these findings were initially interpreted as incidental ischemic lesions, leading the diagnostic pathway toward a vascular etiology. Consequently, early treatment strategies primarily focused on improving cerebral circulation and antiplatelet therapy, without adequate consideration of an immune-mediated mechanism.

Previous studies have shown that AE may initially present with nonspecific prodromal symptoms and subtle or even normal MRI findings, consistent with the early presentation observed in our patient (7, 19, 20). This observation is consistent with the hypothesis that functional abnormalities may precede overt structural imaging changes.

In the present case, inflammatory CSF findings, positive neuronal antibodies, and EEG abnormalities preceded the development of typical MRI abnormalities, further suggesting that immunological testing may provide additional diagnostic information in the early stage when AE is clinically suspected. Therefore, MRI findings alone should not be used to exclude the diagnosis. Instead, a multimodal diagnostic approach integrating EEG, CSF analysis, and neuronal antibody testing is essential, with CSF antibody detection often providing higher diagnostic specificity.

As the disease progressed, MRI findings rapidly evolved into multifocal, predominantly cortical lesions, consistent with the characteristic imaging phenotype of anti-GABA_A_ receptor encephalitis. Importantly, a clear temporal dissociation was observed among immunological, electrophysiological, and imaging markers. Specifically, negativization of CSF antibodies and improvement in EEG abnormalities preceded the resolution of MRI lesions, indicating a temporal lag between immune activity and structural recovery. A similar temporal lag between MRI recovery and clinical improvement has recently been reported by Papi et al. (10), whereas our case further demonstrates the complementary value of integrating EEG and immunological biomarkers during longitudinal follow-up.

The mechanism underlying this temporal delay remains uncertain, although slower resolution of structural injury may contribute. Therefore, while structural MRI remains valuable for longitudinal follow-up and assessment of lesion evolution, it should not be relied upon as the sole indicator for early diagnosis or treatment response evaluation.

Taken together, this case highlights that neuroimaging findings in AE may be subtle, nonspecific, or even normal in the early stage. Overreliance on MRI alone may result in delayed diagnosis. In clinical practice, a multimodal integrative approach is essential, establishing dynamic correlations among clinical presentation, EEG findings, CSF analysis, and antibody testing. Such an approach may improve early recognition and optimize the timing of immunotherapy.

### 3.3 Electroencephalographic features as functional markers of network hyperexcitability

EEG provided important functional information throughout the disease course in this patient (21). In this case, serial EEG recordings revealed diffuse spike-and-slow-wave discharges and frequent epileptiform activity during the acute phase. Notably, the distribution of epileptiform discharges corresponded closely to the anatomical regions of MRI abnormalities. And these EEG abnormalities persisted despite continuous midazolam sedation during the acute phase, further supporting ongoing cortical hyperexcitability. With the initiation of immunotherapy, epileptiform activity gradually decreased, accompanied by restoration of background rhythms. This dynamic evolution was highly consistent with clinical improvement and the decline in antibody titers, suggesting that EEG abnormalities may reflect changes in disease activity rather than merely structural injury in this patient.

Importantly, even after substantial clinical improvement, residual epileptiform discharges were still detectable on EEG, indicating a temporal dissociation between symptom resolution and stabilization of neuronal network activity. This observation supports the concept that electrophysiological dysfunction in AE may precede and persist beyond structural imaging changes.

Taken together, this case highlights the value of serial EEG as a complementary functional biomarker for longitudinal assessment of disease activity and therapeutic response in anti-GABA_A_ receptor encephalitis.

### 3.4 Peripheral and immunological biomarkers of systemic neuroinflammation

Peripheral immune activation is considered a key component in the pathogenesis of AE, including anti-GABA_A_ receptor encephalitis, and elevated IL-6 has been associated with disease severity and clinical outcomes in previous studies (22–24).

In this case, IL-6 levels were markedly elevated during the acute phase and gradually declined following immunotherapy. This trajectory closely paralleled clinical recovery and the reduction of EEG abnormalities, suggesting that IL-6 may provide complementary information regarding immune activity during longitudinal follow-up, although its value should be interpreted in conjunction with clinical and electrophysiological findings.

Furthermore, in this case, systemic autoimmune markers and tumor screening were negative, supporting the notion that the disease represents a primary antibody-mediated autoimmune process rather than a secondary paraneoplastic syndrome or systemic autoimmune disorder.

Overall, this case highlights the complementary value of integrating peripheral inflammatory markers with electrophysiological, imaging, and clinical findings during longitudinal disease monitoring.

### 3.5 Therapeutic response and multimodal concordance of recovery

Current treatment of anti-GABA_A_ receptor encephalitis follows established AE guidelines, with early immunotherapy associated with improved neurological outcomes (23, 25–28).

In current clinical guidelines, plasma exchange is generally regarded as a unified therapeutic approach, without clear differentiation between specific technical modalities. Previous studies have suggested that both TPE and DFPP may confer therapeutic benefits across various autoimmune diseases, with early implementation associated with improved outcomes (29–31). However, in this case, clinical improvement appeared to occur following TPE but not during DFPP. Importantly, this temporal association should not be interpreted as evidence of superior efficacy of one modality over the other, as disease progression, treatment timing, and concomitant immunotherapy may all have influenced the clinical course. Accordingly, this observation should be regarded as hypothesis-generating and requires validation in future studies.

From a mechanistic perspective, TPE and DFPP differ in their modes of immune component removal. TPE involves direct replacement of the patient’s plasma, enabling broad and non-selective removal of circulating plasma proteins, including immunoglobulins, complement factors, and inflammatory mediators (32). In contrast, DFPP employs a two-step filtration system based on molecular size, preferentially removing larger proteins while preserving smaller molecules such as albumin (33). Previous studies have suggested that, compared with conventional TPE, DFPP may have relatively lower efficiency in removing immunoglobulins such as IgG (34).

Given that anti-GABA_A_ receptor encephalitis is primarily mediated by pathogenic IgG antibodies targeting synaptic receptors, a broader and more effective clearance of circulating immune components could theoretically facilitate more rapid reduction of pathogenic antibody levels. This mechanistic difference may partly account for the temporal differences observed in this patient; however, this hypothesis requires validation in future studies.

Besides, despite progressive improvement in structural MRI findings, TSPO-PET continued to demonstrate persistent tracer uptake in bilateral temporo-parieto-occipital regions, which may reflect ongoing neuroinflammatory processes. However, because TSPO-PET lacks complete specificity for microglial activation and no baseline TSPO-PET examination was available for comparison, these findings should be interpreted cautiously.

Overall, antibody negativization, EEG improvement, clinical recovery, and delayed MRI resolution followed distinct temporal trajectories in this patient, illustrating that functional recovery and structural repair may occur on different timelines. Although no conclusions can be drawn from a single case, our findings support the complementary use of clinical assessment, EEG, immunological biomarkers, structural MRI, and molecular imaging during longitudinal follow-up.

## 4 Conclusion and future directions

This case provides longitudinal multimodal evidence illustrating the dynamic evolution of the disease across multiple dimensions. The findings suggest that anti-GABA_A_ receptor encephalitis may involve not only structural inflammatory injury but also dynamic immune-mediated dysfunction of neuronal network excitability.

Importantly, improvement in clinical symptoms and EEG abnormalities clearly preceded the resolution of structural MRI lesions, indicating a significant temporal gap between synaptic functional recovery and structural repair. This observation highlights the limitations of relying solely on structural imaging to assess disease activity and suggests that functional biomarkers, such as EEG and immunological indicators, may complement structural MRI by providing a more real-time reflection of neural network status and disease dynamics.

In addition, this case suggested different temporal clinical responses following TPE and DFPP. Clinical improvement was observed after TPE but not during DFPP. Because this observation is based on a single case, it should be regarded as hypothesis-generating and should not be interpreted as evidence supporting one plasma exchange modality over another.

Future prospective studies integrating multimodal biomarkers are needed to refine disease activity assessment and to advance precision immunotherapeutic strategies.

In conclusion, this case highlights the critical importance of early recognition, timely initiation of immunotherapy, and dynamic multimodal monitoring in preventing irreversible neurological damage and improving clinical outcomes in AE.

### 4.1 Technical details

#### 4.1.1 Autoantibody testing

Autoimmune encephalitis antibody testing was performed at two independent reference laboratories during the disease course. Initial diagnostic testing was conducted at Hangzhou Hongwang Medical Laboratory using a commercial fixed cell-based indirect immunofluorescence assay. The neuronal surface antibody panel included NMDAR, LGI1, CASPR2, GABABR, AMPAR1/2, DPPX, IgLON5, GlyRα1, and GABAA receptor α1, β3, and γ2 subunits. Serum and CSF samples were serially diluted to determine endpoint antibody titers, defined as the highest dilution demonstrating specific immunofluorescence. According to the laboratory protocol, serum testing started at a dilution of 1:10, whereas CSF testing started at 1:1.

In parallel, a CSF TBA was performed using fresh-frozen rat brain sections. The characteristic cerebellar granular layer (synaptic) staining pattern was interpreted together with the CBA findings and the clinical presentation.

Paraneoplastic neuronal antibodies were examined in both serum and CSF using a commercial transfected cell-based indirect immunofluorescence assay provided by Hangzhou Hongwang Medical Laboratory. All results were negative.

Follow-up autoimmune encephalitis antibody testing during treatment was performed at Nanjing Xiansheng Medical Laboratory using a commercial CBA. Follow-up samples were reported qualitatively as positive or negative according to the laboratory protocol, without repeat endpoint titer determination. The results were interpreted in conjunction with the patient’s clinical course.

#### 4.1.2 EEG

Representative EEG tracings were obtained from prolonged EEG monitoring during the acute and peak disease stages (Hospital Days 2 and 7) and from a routine EEG examination during recovery (Hospital Day 20). The acute- and peak-stage EEG recordings were acquired while the patient was receiving continuous midazolam sedation for seizure control, whereas the recovery-stage EEG was performed without sedative medication. EEG recordings were acquired using the international 10–20 electrode placement system with a sampling rate of 250 Hz, a paper speed of 30 mm/s, a low-frequency filter of 1 Hz, a high-frequency filter of 30 Hz, and a sensitivity of 7 μV/mm. All EEGs were qualitatively interpreted by experienced electroencephalographers. Scale bar: 140 μV, 1 s.

#### 4.1.3 MRI

Brain MRI examinations were performed using routine clinical protocols on Philips Ingenia and Siemens MAGNETOM Aera scanners. Representative axial FLAIR, ADC/DWI, and T2-weighted images are shown. Slice thickness ranged from 5 to 6 mm according to the clinical imaging protocol. Imaging findings were qualitatively assessed by experienced neuroradiologists.

#### 4.1.4 TSPO-PET

Brain TSPO-PET imaging was performed approximately 40 minutes after intravenous administration of 18F-DPA714 using a three-dimensional time-of-flight (3D-TOF) acquisition protocol with TrueX reconstruction. Image interpretation was based on qualitative visual assessment. Semi-quantitative analysis and partial volume correction were not performed.

All imaging and electrophysiological findings were interpreted independently by experienced specialists and integrated with the clinical presentation for longitudinal multimodal assessment.

Both serum and CSF samples were tested for the paraneoplastic neuronal antibody panel using a commercial transfected cell-based indirect immunofluorescence assay provided by Hangzhou Hongwang Medical Laboratory according to the manufacturer’s protocol. All antibodies were negative in both serum and CSF.

## Data Availability

The original contributions presented in the study are included in the article/**Supplementary Material**. Further inquiries can be directed to the corresponding authors.
