# Supplementary material for: Multimodal temporal dissociation in anti-GABAA receptor encephalitis: functional recovery precedes structural resolution-a case report
Source: Front Immunol. 2026 Jul 27;17:1857947. doi: 10.3389/fimmu.2026.1857947 (PMC13454428; doi:10.3389/fimmu.2026.1857947)
Supplement: Supplementary file 1 [file SupplementaryFile1.docx]

**Supplementary Materials**

**Figure S1. Representative Immunofluorescence Findings Obtained at the Initial Diagnostic Evaluation (Hospital Day 3).**

(A, B) CBA demonstrating anti-GABA_A_ receptor α1 antibodies in CSF (endpoint titer 1:1) and serum (endpoint titer 1:10), respectively.

(C) Tissue-based assay (TBA) performed using fresh-frozen rat brain tissue sections showing the characteristic cerebellar granular layer (synaptic) staining pattern in the CSF.

(D) Serum TBA was negative.
