# Supplementary material for: Multimodal temporal dissociation in anti-GABAA receptor encephalitis: functional recovery precedes structural resolution-a case report
Source: Front Immunol. 2026 Jul 27;17:1857947. doi: 10.3389/fimmu.2026.1857947 (PMC13454428; doi:10.3389/fimmu.2026.1857947)
Supplement: Supplementary file 2 [file Table1.docx]

**Supplementary Materials**

**Table S1. Peripheral Blood Laboratory Findings during Disease Course**

| Category | Item | Reference Range | On Admission  (2025/9-2025/10-22) | Peak Disease Stage  (2025/10/23) | Post-Immunotherapy  (2025/11/16-11/30) | Clinical Significance |
| --- | --- | --- | --- | --- | --- | --- |
| Inflammatory Markers | WBC (×10⁹/L) | 3.5-9.5 | 6.34 | 23.27 | 8.02 | Exclude infection |
|  | CRP (mg/L) | 0-6 | 1.2 | 1.25 | 21.36 | Systemic inflammation |
|  | PCT (ng/mL) | 0-0.06 | ＜0.04 | 3.303 | 0.207 | Bacterial infection exclusion |
| Autoimmune Screening | ANA | ＜1:100 | 1:100 | - | negative | Exclude systemic autoimmune disease |
|  | ANCA | negative | negative | - | negative | Vasculitis exclusion |
| Thyroid Autoimmunity | TSH(mIU/L) | 0.75-5.6 | 1.11 | - | 1.1 | Endocrine encephalopathy exclusion |
|  | FT4(pmol/L) | 12.8-21.3 | 13.7 | - | 18.4 |  |
|  | TPOAb(IU/mL) | 0-34 | 11.2 | - | 19.8 |  |
|  | TgAb(IU/mL) | 0-115 | 19 | - | 32.1 |  |
| Tumor Screening | CEA(ng/mL) | 0-24 | 15.8 | - | 13 | Paraneoplastic evaluation |
|  | NSE(ng/mL) | 0-16.3 | 12 | - | 14.1 | Neuroendocrine tumor marker |
|  | CYFRA21-1(ng/mL) | 0-3.3 | 4.72 | - | 1.95 | Lung cancer screening |
|  | AFP(ng/mL) | 0-7 | 2.24 | - | 1.3 |  |
|  | CA125(U/mL) | 0-24 | 15.8 | - | 95.8 |  |
| Immune Status | IgG(g/L) | 8.6-17.4 | 9.33 | - | 16.6 | Humoral immune activation |
|  | IgA(g/L) | 1-4.2 | 2.49 | - | 2.8 |  |
|  | IgM(g/L) | 0.3-2.2 | 0.54 | - | 0.28 |  |
| Lymphocyte Subsets | CD3+ T cells(cells/μL) | 876.8-2310.0 | 1010.6 | - | 980.42 | Cell-mediated immunity |
|  | CD4+ T cells(cells/μL) | 455.7-1261 | 696 | - | 771.06 |  |
|  | CD8+ T cells(cells/μL) | 258.9-958.7 | 292.8 | - | 189.39 |  |
|  | CD19+ B cells(cells/μL) | 95.9-412.1 | 206.5 | - | 137.23 |  |
|  | NK cells(cells/μL) | 109.8-780.4 | 260 | - | 59.55 |  |
| Neuroinflammation Biomarkers | IL-6(pg/mL) | 0-5.4 | 13.51 | - | 8.7 | Cytokine storm indicator |
|  | TNF-α(pg/mL) | 0-16.5 | 3.62 | - | ＜2.4 |  |
|  | IL-8(pg/mL) | 0-20.6 | 18.36 | - | 5.3 |  |
