# Supplementary material for: Multimodal temporal dissociation in anti-GABAA receptor encephalitis: functional recovery precedes structural resolution-a case report
Source: Front Immunol. 2026 Jul 27;17:1857947. doi: 10.3389/fimmu.2026.1857947 (PMC13454428; doi:10.3389/fimmu.2026.1857947)
Supplement: Supplementary file 3 [file Table2.docx]

**Supplementary Materials**

**Table S2. Results of Paraneoplastic Neuronal Antibody Testing**

| **Antibody** | **Serum** | **CSF** |
| --- | --- | --- |
| HuB | Negative | Negative |
| HuC | Negative | Negative |
| HuD | Negative | Negative |
| Yo (CDR2) | Negative | Negative |
| Yo (CDR2L) | Negative | Negative |
| Ri (Nova1) | Negative | Negative |
| Ri (Nova2) | Negative | Negative |
| CV2/CRMP5 | Negative | Negative |
| Amphiphysin | Negative | Negative |
| Ma2 | Negative | Negative |
| Ma1 | Negative | Negative |
| Tr (DNER) | Negative | Negative |
| Zic4 | Negative | Negative |
| SOX1 | Negative | Negative |
| GAD65 | Negative | Negative |
| PKCγ | Negative | Negative |
| Recoverin | Negative | Negative |
| Titin | Negative | Negative |

Both serum and CSF samples were tested for the paraneoplastic neuronal antibody panel using a commercial transfected cell-based indirect immunofluorescence assay provided by Hangzhou Hongwang Medical Laboratory according to the manufacturer's protocol. All antibodies were negative in both serum and CSF.
